# Symptomatic Meckel’s Cave Metastasis from Castration-resistant Prostate Cancer Treated with Gamma Knife Radiosurgery

**DOI:** 10.7759/cureus.2839

**Published:** 2018-06-19

**Authors:** Leonid Reshko, Martin K Richardson, Kelly Spencer, William H McAllister IV, Charles R Kersh

**Affiliations:** 1 Medical Education, Riverside Regional Medical Center, Newport News, USA; 2 Riverside Regional Medical Center, Newport News, USA; 3 Radiosurgery, Riverside Regional Medical Center, Newport News, USA; 4 Neurosurgery, Riverside Regional Medical Center, Newport News, USA; 5 Radiation Oncology, Riverside Regional Medical Center, Newport News, USA

**Keywords:** meckel’s cave, prostate cancer, metastatic prostate cancer, skull base tumor, radiosurgery, gamma knife, stereotactic radiosurgery, srs, palliative care, trigeminal nerve palsy

## Abstract

Prostate cancer commonly spreads to the axial and appendicular skeleton, but metastases to the brain parenchyma or skull base are uncommon. In the cases that this happens, the symptoms are usually associated with disease involving the orbit. Metastasis to the Meckel’s cave causing trigeminal nerve palsy is an exceedingly rare entity. We are presenting a case of this in a man with metastatic castration-resistant prostate cancer. Metastatic prostate cancer to the Meckel's cave is extremely uncommon, and there is no standard of care. Radiation therapy, especially radiosurgery, is increasingly recognized as an excellent alternative to surgery for lesions in the Meckel’s cave and intracranial/skull base prostate cancer metastases. Gamma Knife radiosurgery (Elekta, Stockholm, Sweden), in particular, has been reported to achieve local control close to 90% for calvarial and skull base metastases with few side effects and requires only one treatment. Our patient’s 1.4 x 1.0 x 1.3 cm metastatic prostate cancer lesion in the Meckel's cave was treated with Gamma Knife to 22 Gy with good treatment response including rapid improvement in his symptoms and no side effects. We review the scarce literature documenting cases of prostate cancer metastatic to the brain or skull base and the only two other documented cases of prostate cancer metastasis the Meckel’s cave neither of which was treated with radiotherapy.

## Introduction

Prostate cancer has a propensity for metastasis to the bone. However, metastasis to the brain parenchyma or skull base is relatively uncommon. In the rare cases that this does happen, most of the signs and symptoms are due to the disease involving the orbit and supratentorial space [1-3]. Metastasis specifically to the Meckel's cave is even rarer with only two documented cases [4-5]. The Meckel’s cave is a dural recess in the posteromedial portion of the middle cranial fossa and is the location of the trigeminal ganglion [6]. There is no standardized treatment for this disease. National Comprehensive Cancer Network (NCCN) and other major prostate cancer treatment guidelines do not address this specific type of bone metastasis. The options include surgery, chemotherapy, and/or radiation therapy [7]. Radiation therapy offers potential to address metastatic intracranial tumors for which surgical resection would be prohibitively morbid [8-9]. While surgery has been used successfully [4], radiation therapy is often a more appropriate modality and is increasingly more frequently used given the patients' poor prognosis with mean overall survival as ranging from 1 to 13 months [10]. Cranial nerve involvement, in particular, indicates a particularly poor prognosis [1]. Radiosurgery such as Gamma Knife (Elekta, Stockholm, Sweden) has been a promising modality due to more precise targeting, normal tissue sparing, and less frequent physician visits since only a single treatment is required [7, 10]. As they have never been published, reports documenting the feasibility of Gamma Knife radiosurgery in prostate cancer metastatic to Meckel’s cave are needed.

## Case presentation

The patient was a 64-year-old male with a history of diffusely metastatic castration-resistant prostate cancer. The disease was initially discovered due to an elevated prostate-specific antigen (PSA) of 4-5 in 2005 with two negative prostate biopsies. His PSA level went up to 9.0 and eventually to 78.4. Magnetic resonance imaging (MRI) at that time revealed a large prostate with the disease in the left pelvic lymph node, extracapsular extension, and a left hip pathologic fracture. A pelvic lymph node biopsy revealed prostate adenocarcinoma in June 2016. The patient was started on triptorelin in June 2016, followed by leuprolide acetate, bicalutamide, and denosumab soon after. Palliative radiation therapy to the left hip and femur and open reduction internal fixation were performed. In spite of this, his PSA continued to rise in December 2016 and a positron emission tomography/computed tomography (PET/CT) scan revealed diffuse osseous disease in the axial and appendicular skeleton and pelvic lymphadenopathy. Casodex was withdrawn, and the patient was started on abiraterone and prednisone in April 2017. Unfortunately, he developed a new osseous right femoral lesion which was treated with palliative radiation therapy. A PET/CT scan revealed further progressive osseous disease and pelvic lymphadenopathy. Abiraterone was stopped and docetaxel was initiated in addition to prednisone in September 2017. A bone marrow biopsy revealed extensive involvement of metastatic prostate cancer. Denosumab was stopped and enzalutamide was started in December 2017. In January 2018, enzalutamide was stopped due to intolerance.

**Figure 1:**
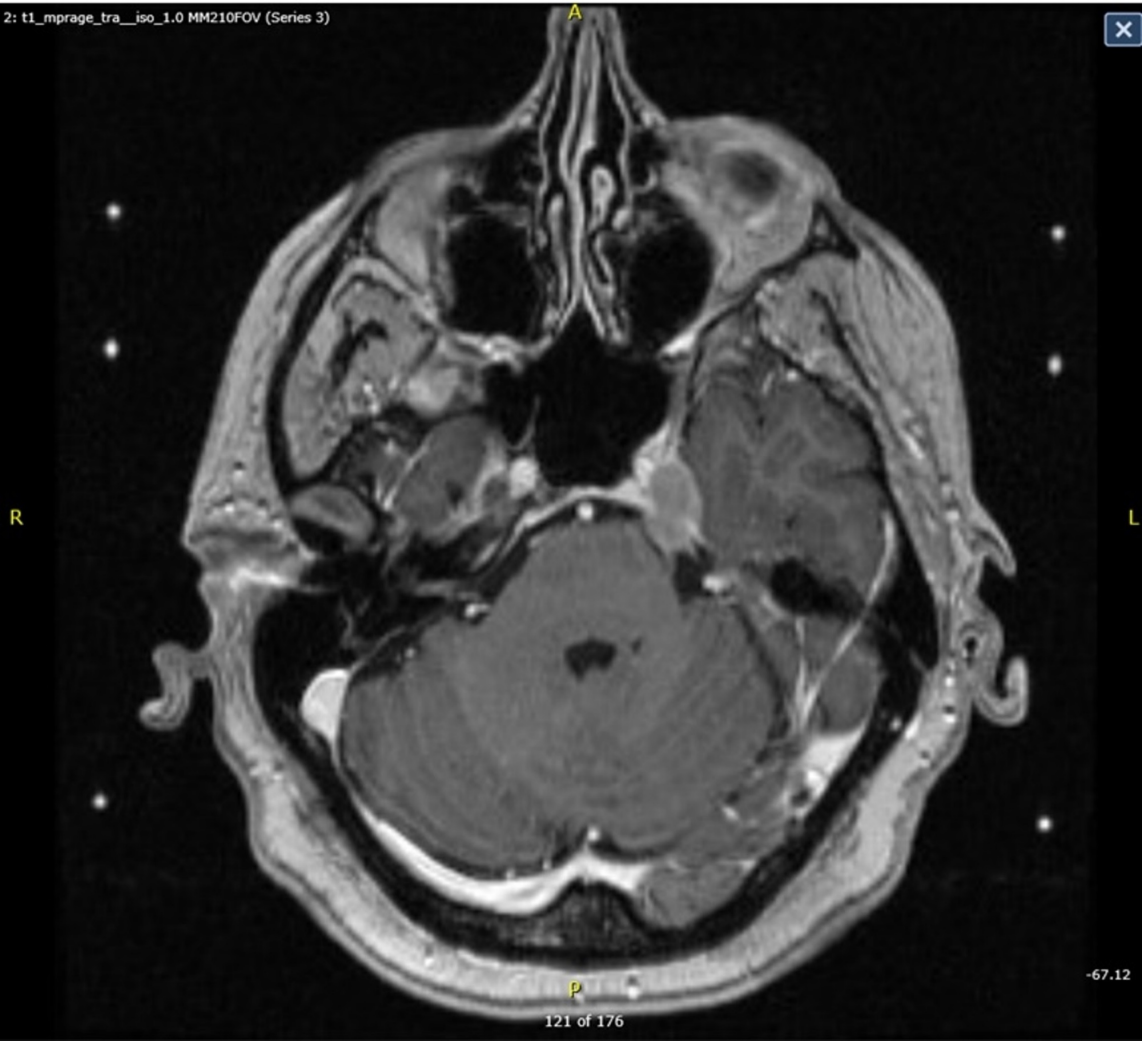
Pre-treatment Gamma Knife MRI MRI: magnetic resonance imaging

He presented to us one year and seven months after his pathologic diagnosis for treatment of a single lesion measuring 1.4 x 1.0 x 1.3 cm in the Meckel’s cave contiguous with the left cranial nerve V with some extension into the prepontine cistern. This was consistent with being metastatic cancer on radiologic appearance as illustrated in Figure 1. The patient developed left forehead and cheek numbness two weeks prior to presenting to our clinic. This led to difficulty with eating secondary to numbness. Objectively, there was numbness in the left-sided ophthalmic branch of the cranial nerve V.  His other medical problems included well-controlled diabetes type 2, hypertension, depression, and various complications of his cancer treatment which impaired his functional status including leg numbness since his deep vein thrombosis (DVT) status post inferior vena cava (IVC) filter placement, cancer-related bone pain on oxycodone, fatigue, tingling and numbness in his hands, pancytopenia and osteonecrosis of the jaw. Given that this patient had widespread castration-resistant prostate cancer with a metastatic lesion causing cranial nerve dysfunction, he had a very poor prognosis. Treatment with radiotherapy was initiated without a pathologic confirmation to produce the most rapid symptomatic relief without morbidity and possible mortality associated with biopsy and surgical resection.

**Figure 2:**
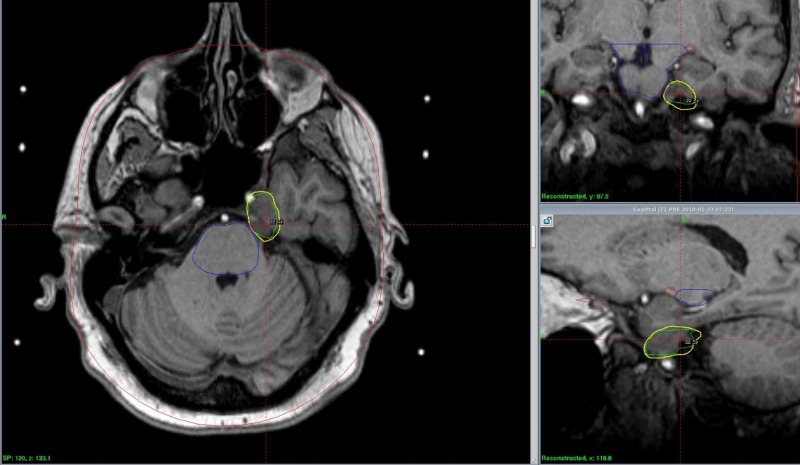
Gamma Knife treatment plan with the tumor GTV outlined in green, 50% isodose line in yellow, and brainstem in blue GTV: gross total volume

A radiation therapy dose of 22 Gy was given to the 50% isodose line via 17 shots, delivered via Leksell Gamma Knife produced by Elekta (Stockholm, Sweden) to the gross tumor volume (GTV) of 2.11 cc with palliative intent. The treatment plan is shown in Figure 2. Tumor coverage was 100%. Maximum dose was 44 Gy. The patient’s head was immobilized using a Gamma Knife Perfexion (Elekta, Stockholm, Sweden) head frame with metal pins. Constraints for all organs at risk were met. There were no complications from the procedure.

On a one-month follow-up, the patient’s facial sensation has improved, leading to a better ability to eat. No additional cranial nerve dysfunction or other neurologic deficits were noted. No side effects attributable to Gamma Knife radiosurgery were observed. The patient decided to stop undergoing systemic chemotherapy soon after his last follow-up visit with us stating that chemotherapy caused fatigue and that the infusion center visits took up too much of his time. The patient elected to go home on hospice care. Unfortunately, the patient died three months after the Gamma Knife procedure. No new neurologic deficits or Gamma Knife side effect were recorded.

## Discussion

Metastatic prostate cancer to the Meckel’s cave

Only one case report and one case series clearly document cases presenting this condition. In the first, there was a patient with a 3.4 x 4 cm skull base tumor with its epicenter located in a completely obliterated Meckel’s cave. The patient had worsening gait, left-sided facial weakness, disorientation, speech, and numbness from pontomesencephalic cranial nerve and temporal lobe compression. Biopsy, angiography, and surgical resection via petrosal approach were performed. The lesion was determined to be metastatic prostate adenocarcinoma. No additional metastatic lesions or whether there was a mass at the primary site were reported. At two months, all of the patient’s signs had improved. No further information about the outcome of this case was reported [4]. In the second case, it was reported that a patient had a facial palsy with cranial nerve (CN) V and XII involvement by undifferentiated carcinoma, presumably from the prostate gland, but no treatment or details of radiological findings were reported [3].

Diagnosis

While biopsy or pathology from a resection specimen is ideal for diagnosis, it is not always readily accessible especially in a case when it would lead to significant morbidity and possibly mortality. In a large retrospective study, the rate of mortality from a biopsy of cavernous sinus and Meckel’s cave lesions was 2%, permanent cranial nerve deficits occurred in 4% of cases, and 14% of the biopsies were non-diagnostic [5]. The yield of the tumor biopsy is particularly low in patients whose tumor appearance on imaging is consistent with clinical presentation. In these cases, treatment without a tissue diagnosis may be appropriate [5]. This is particularly so in our patient in whom his tumor caused cranial nerve dysfunction leading to a very poor prognosis and for whom expedited palliation was needed.

Treatment

While surgery has been used successfully [4], radiation therapy is often a more appropriate modality given the patient’s poor prognosis with mean overall survival ranging from 1.8 to 13 months [10] for skull base metastases and one month median survival for adenocarcinoma metastatic to the brain in the largest retrospective study [2]. A review of skull-base metastases in 2005 found a median survival of 31 months for skull base tumors, but the survival was only five months for patients with cranial nerve palsies, such as the case presented here. The prognosis for patients with metastases to the Meckel’s cave is more difficult to estimate, but a patient with cranial nerve palsy is likely near the end of life. Surgical resection in this area carries a high risk of morbidity and chance of mortality given the procedural complexity and the patients' medical co-morbidities and suboptimal functional status [10]. Radiation therapy has been reported as a successful treatment modality for this disease, and it offers the ability to address metastatic intracranial tumors for which surgical resection would be prohibitively morbid [9-10]. Radiosurgery is particularly promising due to its more precise targeting, normal tissue sparing, and less frequent visits to the physician required due to fewer fractions of ionizing radiation utilized [10]. In particular, Gamma Knife radiosurgery has been shown to be effective in a retrospective study with a local control rate of 88.9% and an overall survival of 36 months in patients with calvarial and skull base metastases with minimal side effects, albeit no metastatic prostate cancer patients were included in the study [7]. Gamma Knife radiosurgery was successfully employed in our case with no procedural complications or side effects, and it resulted in rapid symptomatic relief.

Limitations

As is true for all case reports, the generalizability of this information is limited. The outcome of Gamma Knife radiosurgery for prostate cancer metastatic to Meckel’s cave is difficult to estimate accurately from this information alone. However, given the extreme rarity of this condition and the fact that Gamma Knife radiosurgery has never been reported as a treatment, this case report is an important contribution to this particular topic.

Future Directions

Due to a paucity of data, additional reports are needed to assess the feasibility of Gamma Knife radiosurgery in prostate cancer metastatic to Meckel’s cave causing cranial nerve dysfunction. If this modality is shown to be successful in multiple reports, then it should be strongly considered as a standard of care for these patients given its excellent local control, rapid efficacy, and low side effect profile.

## Conclusions

The optimal treatment for castration-resistant prostate cancer metastatic to Meckel’s cave fthat causes trigeminal nerve deficits is uncertain due to its extreme rarity. However, in conjunction with studies that evaluated Gamma Knife and other radiosurgery modalities in skull base tumors and brain parenchymal metastases, it appears that Gamma Knife offers excellent local control, rapid symptom palliation, and a low side effect profile. Gamma Knife is moreover less invasive than surgery and is less demanding for the patient than standard fractionated radiation therapy while offering superior normal tissue sparing and tumor targeting.
